# The Association of Neighborhood Changes with Health-Related Quality of Life in the Women’s Health Initiative

**DOI:** 10.3390/ijerph19095309

**Published:** 2022-04-27

**Authors:** Benjamin W. Chrisinger, Sparkle Springfield, Eric A. Whitsel, Aladdin H. Shadyab, Jessica L. Krok-Schoen, Lorena Garcia, Shawnita Sealy-Jefferson, Marcia L. Stefanick

**Affiliations:** 1 Department of Social Policy and Intervention, University of Oxford, Oxford OX1 2ER, UK; 2 Department of Public Health Sciences, Loyola University Chicago, Chicago, IL 60660, USA; 3 Department of Epidemiology, Gillings School of Global Public Health, University of North Carolina-Chapel Hill, Chapel Hill, NC 27599, USA; eric_whitsel@med.unc.edu; 4 Department of Medicine, School of Medicine, University of North Carolina-Chapel Hill, Chapel Hill, NC 27599, USA; 5 Herbert Wertheim School of Public Health and Human Longevity Science, University of California, San Diego, CA 92093, USA; ahshadya@health.ucsd.edu; 6 School of Health and Rehabilitation Sciences, Ohio State University, Columbus, OH 43210, USA; jessica.schoen@osumc.edu; 7 Department of Public Health Sciences, School Medicine, University of California, Davis, CA 95616, USA; lgarcia@ucdavis.edu; 8 College of Public Health, Ohio State University, Columbus, OH 43210, USA; sealy-jefferson.1@osu.edu; 9 Stanford Prevention Research Center, Stanford University School of Medicine, Stanford, CA 94305, USA; stefanick@stanford.edu

**Keywords:** women’s health initiative, neighborhoods, quality of life, neighborhood change

## Abstract

Longitudinal studies can help us understand the effects of long-term neighborhood changes, as these can capture individual self-appraisal of current and future circumstances. We analyzed the association between neighborhood changes and health-related quality of life (HRQoL) outcomes among older women from the Women’s Health Initiative (WHI) study. We used a subset (*n* = 49,254) of the longitudinal WHI dataset of female participants, aged 50–79 at baseline, recruited from 40 clinical centers across the U.S. beginning in 1993. Two HRQoL outcomes were explored: self-rated quality of life (SRQoL), and physical functioning-related quality of life (PFQoL). We used U.S. census tract-level changes in median household income between the 2000 census and 2007–2011 American Community Survey to classify neighborhoods as “upgrading,” “declining,” or “stable.” Multi-level models were used to identify significant associations between neighborhood change and HRQoL outcomes over time. Compared to participants residing in upgrading neighborhoods, participants in stable and declining neighborhoods reported significantly lower PFQoL. A significant interaction was observed with income such that the effect of neighborhood change was greater at lower levels of income.

## 1. Introduction

Health-related quality of life (HRQoL) refers to a person’s or group’s perceived physical and mental health over time [1]. The construct often encompasses a self-appraisal of satisfaction with life and outlook toward the future. Measures of HRQoL do not include specific reference to disease states or illnesses, which demonstrate its consistency with more holistic conceptualizations of health promoted by the World Health Organization and other national and international health agencies [2,3]. As a measure of perception, HRQoL is embedded within individual circumstances, feelings, and attitudes and the interactions and trade-offs between them [3]. Measures of quality of life vary from simple, single-question assessments to more complex multi-item constructs. Proponents of HRQoL measures argue that these measures provide a useful high-level indicator of change over time or in response to an intervention, allowing for targeted improvements to various health services [2].

Though they are not precise clinical diagnostics, studies have found these self-appraisals of mind and body to be significantly associated with poor health outcomes, including cardiovascular disease, as well as cause-specific and all-cause mortality risk in older women [4,5,6,7,8,9,10,11,12,13]. These associations are echoed in the Women’s Health Initiative (WHI) [11], the primary population for this paper, which analyzes a subset of WHI participant data. Thus, while self-reported HRQoL measures primarily describe an individual’s perception of themselves rather than any diagnosed condition or illness, they can also illuminate broader clinical issues.

**Figure 1:**
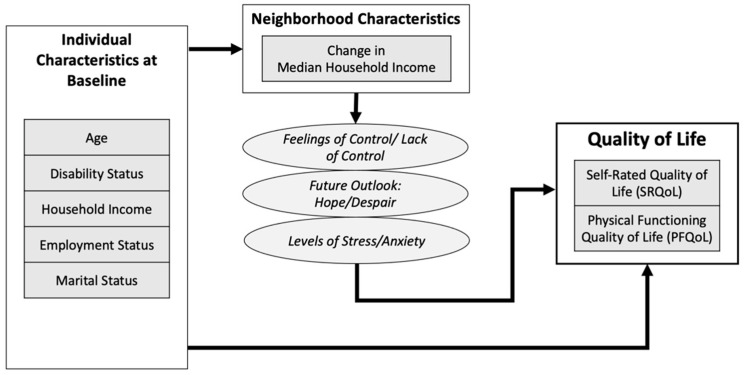
Logic model for relationships between neighborhood change and quality of life outcomes.

In numerous cross-sectional studies, neighborhood-level factors have also been strongly associated with individuals’ quality of life [14,15,16]. Still, the dynamism of neighborhood environments challenges the internal validity of point-in-time estimates of neighborhood conditions, motivating scholars to emphasize the importance of representing change in analyses of place and health [17]. Neighborhood infrastructure (e.g., sidewalks, lighting, parks, transport, etc.), demographics (e.g., age, race, ethnicity, disability status, etc.), and socioeconomic conditions (e.g., employment status, educational attainment, access to health resources, etc.) can change over time and reflect broader regional to national social, economic, and political changes (e.g., the breadth and depth of homeownership assistance programs, prevalence of racist attitudes and practices, etc.). Given the importance of contextualized self-appraisal to HRQoL, it is important to consider how *changing* neighborhood environments might affect these assessments beyond more common static measures. In Figure 1, we suggest a logic model for how individual-level quality of life could be influenced by neighborhood-level changes.

Evidence on neighborhood change and quality of life is limited although the extensive upheavals following the 2008 U.S. housing crisis and Great Recession have provided grounds for retrospective evaluations. Some evidence indicates how the neighborhood-level impacts of the economic crisis were unevenly distributed, with lower-income neighborhoods losing more of the economic gains of the previous decade and becoming less able to “bounce back” afterward compared to higher-income communities [18,19]. At the ground level, communities faced unprecedented disruptions as a result of foreclosures, whereby neighborhoods lost resident homeowners who were often not replaced; instead, in many areas, high rates of vacancy resulted from the surge in foreclosures [20,21]. Beyond the destabilization of social networks and resources that high vacancy rates might produce, crime was also significantly associated with foreclosure rates during this period [22]. Recognizing the potential for these kinds of negative feedback loops to further undermine and destabilize neighborhoods, the U.S. Congress allocated USD 7 billion to the Neighborhood Stabilization Program in July 2008, with the hope of slowing an accelerating crisis felt by communities around the country [23].

Several studies and systematic reviews have demonstrated the neighborhood-level effects of foreclosures and their deleterious impact on individual psychological and behavioral health outcomes [13,24,25,26]. For example, data from the National Social Life, Health, and Aging Project revealed an association between depressive symptoms, default notices, and the prevalence of bank-owned properties, a result of homeowners falling behind on mortgage payments and homes ultimately being repossessed by lenders [27]. Settels found city-level foreclosure rates and declines in median home prices to be associated with increases in depressive symptoms among older adults during the Great Recession even after adjusting for any individual-level financial losses, suggesting possible community-level mechanisms [28]. Yet, others have found no evidence of associations between foreclosures with downstream health outcomes. For instance, Downing and colleagues found no association between changes in neighborhood foreclosure rates and glycemic control among a population of continuously insured people with diabetes [25] although others have cautioned against broadly generalizing from these findings [29].

More broadly, investigating neighborhood changes can provide important historical social context for understanding present-day disparities in HRQoL [30]. Adverse effects from the housing crisis spread unevenly across racial, ethnic, and socioeconomic lines and in some cases exacerbated existing geographic disparities. Studies have shown that, as a result of the housing crisis, neighborhoods with high ethnic integration and with large African American or Hispanic populations experienced higher foreclosure rates than predominantly white neighborhoods [31,32]. Disproportionately high foreclosure rates in predominantly African American and Hispanic neighborhoods displaced residents and destabilized communities and contributed to significant changes in the social, economic, and cultural contexts of these neighborhoods [31,32]. Further, these changes may have worsened existing patterns of neighborhood racial and ethnic segregation, isolation, and poor health outcomes [30,31,33]. Evidence suggests communities with high concentrations of historically marginalized racial and ethnic minorities experience lower access to socioeconomic resources (i.e., opportunities for employment and education) and health resources (i.e., full-service supermarkets and safe streets), which may have further adverse effects on individual-level HRQoL [34,35,36]. Moreover, evidence suggests that women experience disproportionately higher rates of risky lending compared to their male counterparts. Some evidence suggests similar practices in older adults (aged 50+) [37]. Thus, there is a broader need for research that considers the interconnected nature of race(ism), class(ism), sex(ism), age(ism), and how these interdependent systems of oppression are linked to housing inequities (e.g., discriminatory lending and foreclosure) as well as the need for women-centered research studies focused on place [38].

In this study, we used data from the Women’s Health Initiative (WHI) observational study cohort coupled with neighborhood-level indicators from the U.S. Census Bureau [39] to examine neighborhood changes in relation to quality of life outcomes. Additionally, given well-documented patterns of residential segregation by income and race, we explored possible interaction effects with participants’ household incomes and race. To our knowledge, the WHI offers the most extensive longitudinal assessment of geographic and quality of life among an exclusive sample of socio-demographically diverse older women. Considering that the WHI enrollment occurred between 1993 and 1998, it is likely that over the past two decades, many participants witnessed significant changes in their neighborhoods in terms of median housing values, related factors such as sociodemographic composition, and the physical and social environment. While national datasets, such as the decennial U.S. census, offer descriptions of neighborhood-level socio-demographic changes (i.e., where changes occurred and the direction of the changes), datasets such as WHI enable more detailed investigations of the relationships between neighborhood change and health outcomes.

## 2. Materials and Methods

### 2.1. Design and Study Population

The primary aim of this study was to describe neighborhood change within WHI participant neighborhoods (defined here as “U.S. Census tracts”). Using an adaptation of Landis’ “double decile difference” methodology to identify stable, declining, and upgrading neighborhoods [39], we examined relationships between quality of life (as measured by the SF-36 Rand Quality of Life scale [40]) and neighborhood changes using partial and full adjustments for individual-level covariates.

The WHI is a large prospective study investigating causes of morbidity and mortality among postmenopausal women. Details on the WHI clinical trials (CT) and observational study (OS) rationale, designs, and consent have been reported elsewhere [41,42,43]. Briefly, 161,808 postmenopausal women aged 50 to 79 years at baseline provided written informed consent to participate in at least one of two clinical trials (Menopausal Hormone Therapy and/or Diet Modification, with the opportunity to enroll in a trial of calcium and vitamin D supplementation a year later) or the OS between 1993 and 1998 at 40 clinical sites across the U.S. through 2005, at which time active study participants were invited to continue participating for five years at their respective clinical centers (2005–2010). Data on the main HRQoL outcomes, including self-rated physical functioning and quality of life, and other sociodemographic and health-related covariates were all collected at baseline. Written informed consent was obtained from all study participants at initial enrollment and again in 2005 for each of the trials they chose to join or the OS with approval by the institutional review boards of the 40 participating institutions across the U.S. In 2010, women who were still actively participating were invited to continue ongoing follow-up in the WHI Extension Study (2010–present) through four WHI regional centers and the WHI Coordinating Center at the Fred Hutchinson Cancer Research Center, which assumed the role of IRB of record for each participant, with Institutional Review Board approval obtained by all institutions involved.

### 2.2. Measures

#### 2.2.1. Neighborhood Change 

Data from WHI, U.S. Census. and American Community Survey (ACS) were used to approximate neighborhood change. Using their mailing addresses, which were updated at least biannually, neighborhood data were assigned to all participants’ clinical and survey-based data points based on their temporal proximity to one of four U.S. Census Bureau data products: 2000 census, 2005–2009 ACS, 2006–2010 ACS, and 2007–2011 ACS. Thus, a maximum of four neighborhood observations were expected for each participant, with higher numbers of observations used to identify individuals who moved to a new census tract. For the purpose of anonymization, the specific dates of survey responses are not typically available as part of the WHI dataset; however, all neighborhood census data were assigned to participants in the same manner, ensuring that observations assigned to census 2000 neighborhood data preceded those assigned to ACS 2005–2009 data, etc., such that each observation cannot be assigned to more than one census product.

Here, we focused on the relative change in median household income between census observations as a proxy for broader neighborhood processes, allowing for comparisons between and across (1) different baseline levels of economic status and (2) different change patterns: stable, upgrading, and declining. We modified a relatively simple method described by Landis (2016), “double decile difference”, to consider the broader context of neighborhood change and its magnitude. Under Landis’s approach, each census tract was given a decile ranking within its metropolitan area according to median household income for a particular time period (Landis computed decile rankings using data from the 2000 and 2010 U.S. census). Tracts that increased in decile ranking between time periods by more than two were defined as “upgrading”, tracts that decreased in ranking by more than two were defined as “declining”, and tracts that changed by less than two decile ranks were defined as “stable.”

Like Landis, other researchers commonly use the median household income census variable as an indicator of neighborhood change [40,41,42]. Relevant variables describing housing, such as average rents and building age and size and demographic factors, such as educational attainment and racial and ethnic diversity, are also frequently considered [40,43]. Some studies go further by supplementing census statistics with local administrative datasets and primary data collection [40,43,44,45]. With each added variable or data source, new assumptions are made about the relevance of observed changes in one place versus others; furthermore, many of these variables (e.g., education and income) are known to be strongly correlated. Thus, important questions arise about how to generally and parsimoniously describe local changes at a national scale.

Though the double decile difference measure paints a very high-level picture of neighborhoods without qualitative nuance, Landis suggested that is has three main benefits for analysts interested in characterizing local change: (1) its applicability across metropolitan areas, which is particularly useful in national studies; (2) its focus on “substantial” neighborhood changes, which hedges against overinterpretation of smaller shifts; and (3) by using decile rankings, its ability to avoid having to define exactly how much household income change is reflective of neighborhood processes [39].

**Figure 2:**
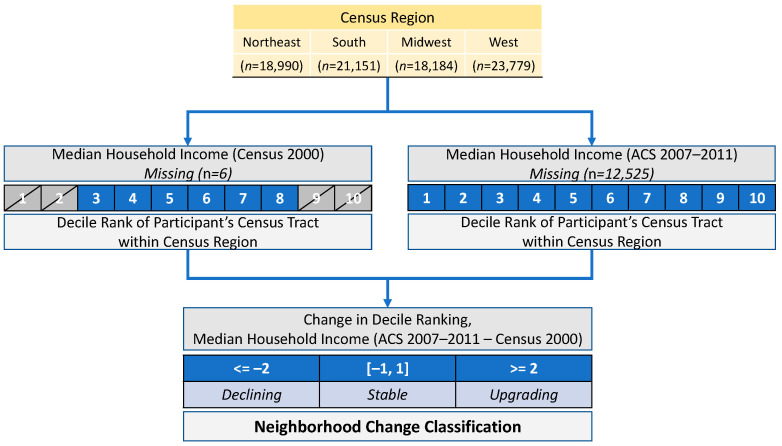
Diagram of neighborhood change variable derivation.

In our modified double decile difference approach (see Figure 2), U.S. census regions (*n* = 4) were used to assign decile ranks to participants based on the median household income of their census tract. Decile rankings within regions were calculated using data from census 2000 (the first available observation and before the Great Recession of 2008 and ACS 2007–2011 (the last available observation)). Baseline decile groups were used to create a continuous variable for relative neighborhood median income closest to the time of enrollment. Figure 2 illustrates how these neighborhood change classifications were defined and derived.

#### 2.2.2. Sociodemographic Characteristics

The self-reported sociodemographic variables that were assessed at baseline included: age (50–54 (reference group), 55–59, 60–69, 70–79); race (White, African American/Black, Asian, and American Indian/Alaska Native, Native Hawaiian or Other Pacific Islander, more than one race); ethnicity (binary: Hispanic versus non-Hispanic), education level (<high school, high school/general education diploma or less, vocational or training school diploma, college or baccalaureate degree, some post-graduate education or higher); annual household income (<USD 20,000 per year (reference group), USD 20,000–USD 34,999, USD 35,000–USD 49,999, USD 50,000–USD 74,999, USD 75,000+); marital status (never married, divorced or separated, widowed, presently married/married-like relationship (reference)); employment status (binary: not employed full-time (reference)); and disability status (binary: not disabled (reference)).

#### 2.2.3. Quality of Life Outcomes

The RAND 36-Item Short-Form Health Survey, the HRQoL measure employed in WHI, included subscales for self-rated quality of life (SRQoL) and physical functioning quality of life (PFQoL) [46,47,48]. SRQoL was assessed with a single item—“Overall, how would you rate your quality of life”—and could be answered on an ordinal scale from 0 to 10. PFQoL was comprised of nine items. For example, one item asked, “Does your health now limit you in vigorous activities, such as running, lifting heavy boxes, or strenuous sports? And, if so, how much?” Response options ranged from 0 = “No, not at all” to 4 = “Yes, limited, a lot.” PFQoL scores ranged from 0–100, with a higher score indicating a more favorable health state in regard to physical functioning [47]. In the WHI dataset, SRQoL and PFQoL subscales were assessed most frequently compared to other HRQoL subscales; the frequency of measurement varied between participants and is reported in the Results (Section 3.1).

#### 2.2.4. Inclusion and Exclusion Criteria 

**Figure 3:**
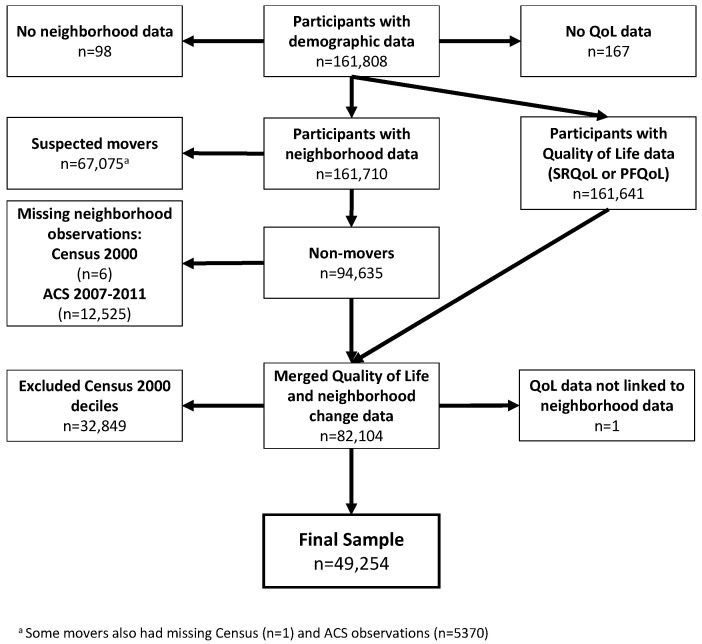
Participant flow diagram.

The original WHI dataset (*n* = 161,808) was reduced according to several inclusion/exclusion criteria. First, participants without any neighborhood (*n* = 98) or quality of life (no SRQoL or PFQoL observations, *n* = 167) data were removed. Next, possible movers were removed. The current WHI dataset does not include a variable to identify whether or not a participant has moved; however, we approximate mover status by exploiting the multiple observations over time which are linked to the 2000 census, 2005–2009 ACS, 2006–2010 ACS, or 2007–2011 ACS. By identifying changes in neighborhood data from the same census product at the participant level, we identified and removed likely movers (e.g., multiple values for median household income are reported within the same census product). Though an indirect approximation, the rate of moving identified within this dataset (*n* = 67,075, 41%) is close to national rate from 2000–2011 (38%) for adults aged 50 and older, as identified by the Panel Study of Income Dynamics [49]. Next, participants without neighborhood data from the 2000 census (*n* = 6) and 2007–2011 ACS (*n* = 12,525) were removed, as these were required to calculate neighborhood change. Finally, we removed participants in the lowest and highest two neighborhood income deciles at baseline (see Figure 2), as downward/upward changes would not have been observable via the double-decile method (i.e., it would be impossible to define a “declining” change in the lowest two baseline deciles). A flow diagram (Figure 3) illustrates the process of achieving the final sample.

### 2.3. Statistical Analysis

To investigate the association between neighborhood change and HRQoL, we used the *lme4* R package [50] to fit a series of multilevel regression models for both SRQoL and PFQoL outcomes, adjusting for sequentially added covariates. The covariates included in all models were pre-specified and included sociodemographic characteristics (age and disability status); socioeconomic factors (annual household income, marital status, and employment status); and neighborhood median household income at baseline. A binary variable accounting for the study design (i.e., WHI clinical trial or observational study) was also included to account for differences in health status at enrollment (e.g., CT participants were screened for health problems, while OS participants were not). Additionally, all models were fit as unconditional growth models using a participant-specific time variable to order their QoL observations and assess within-participant variability. In all models, time was used as the level 1 unit and participants as level 2. To account for the fact that both QoL measures could decrease over time in a non-linear fashion, second- and third-degree raw polynomial time variables were also explored in minimal models that only included time as a covariate, and model fits were compared using ANOVA. In both models, a third-degree polynomial was selected based on ANOVA comparisons.

Following these minimal models (Model 0), a model including only neighborhood change categorical variable was fit (Model 1), with subsequent models adding individual sociodemographic characteristics (Model 2) and tract-level decile rank of median household income at baseline (Model 3). Confidence intervals (alpha = 0.05) were calculated using 500 bootstrap samples generated with the *lme4* package. While stable-classified neighborhoods served as the reference group in all models, estimated marginal means (EMM) were also calculated, and pairwise comparisons were performed to assess significant differences between all neighborhood classifications.

To explore whether the association differed among racial and ethnic groups, income levels, and baseline neighborhood-level income, we also added these as interaction terms to fully adjusted models. Using the *car* package [51], ANOVA was used to jointly test the null hypothesis that the coefficient on the statistical interaction terms was equal to 0.

Analyses were performed using R statistical programming language (Version 3.6.3) in RStudio (Version 1.3.959).

## 3. Results

### 3.1. Description 

Differences in the distribution of demographic characteristics (greater than 5%) between included and excluded participants were observed for several variables: age (18% of included participants were aged 70–79 at baseline versus 24% of excluded participants), income (12% of included participants had household incomes under USD 20,000 versus 17% of excluded participants), and marital status (67% of included participants were presently married or in a marriage-like relationship versus 60% of excluded participants). These participants collectively represented 527,082 observations of SRQoL (mean = 10.7 per participant, range 1–23) and 487,664 observations of PFQoL (mean = 9.9 per participant, range 1–23). For descriptions of additional sociodemographic and health factors in the context of neighborhood change, see Table 1. Of the included participants, a large majority lived in stable neighborhoods *(n* = 37,879; 76.9%), while fewer resided in declining (*n* = 6022; 12.2%) or upgrading (*n* = 5353; 10.9%) neighborhoods; these proportions are roughly similar to those for Landis’ neighborhood classifications of Census tracts in the 70 largest metropolitan areas from 1990–2010 (71.3% stable, 18.0% declining, 10.7% upgrading).

In terms of demographic characteristics and neighborhood change classification, significant differences were observed by age, race, household income, educational attainment, marital status, and the census region of residence (see Table 1). Of these differences, the largest was the proportion of Black/African American women who lived in declining neighborhoods (*n* = 816, 22% of Black/African American participants) compared to White (*n* = 4889, 12% of White participants) and Asian women (*n* = 126, 7% of Asian participants). Only 67% of Black/African American women lived in stable neighborhoods compared with 78% of White women and 79% of Asian women. The proportion of Black/African American women residing in upgrading neighborhoods was similar to other racial groups.

On average, mean participant SRQoL scores for those residing in upgrading neighborhoods (mean = 8.00, SD = 1.59) was slightly higher than those in stable (mean = 7.97, SD = 1.60) and declining (mean = 7.97, SD = 1.61) neighborhoods (see Table 2). For PFQoL scores, upgrading neighborhoods also had the highest averages (mean = 74.3, SD = 25.0) compared to stable (mean = 73.1 SD = 25.4) and declining (mean = 72.9, SD = 25.6) neighborhoods.

### 3.2. Unadjusted Findings 

In unadjusted models, women who lived in upgrading neighborhoods had significantly higher PFQoL (β = 1.04; CI: 0.55, 1.58) compared to those residing in stable neighborhoods (see Table 3). Similarly, women residing in declining neighborhoods had significantly lower PFQoL scores than those in upgrading neighborhoods (estimate = −1.35, *p* = 0.001). No significant differences in PFQoL were observed between stable and declining neighborhoods or between any neighborhoods for the SRQoL outcome.

### 3.3. Fully Adjusted 

Given similar baseline sociodemographic factors (i.e., age, income, marital status, employment status), health factors (i.e., disability status), and neighborhood factors (i.e., baseline median household income), no significant differences in SRQoL were observed between neighborhood change classifications. Additionally, interaction terms between the categorical neighborhood change variable and race, ethnicity, household income, and baseline neighborhood income decile (measured in separate models) were not statistically significant.

Compared to women living in stable neighborhoods, PFQoL was 1.18 (95% confidence interval: 0.68, 1.82) units higher in fully adjusted models among women who lived in upgrading neighborhoods. Similarly, women who lived in declining neighborhoods had significantly lower levels of PFQoL compared to those residing in upgrading neighborhoods (estimate = −1.64, SE = 0.33 *p* < 0.001). A significant interaction was observed between neighborhood change and participant household income in which the differences between neighborhood change categories were most pronounced among lowest-income group (the reference category), with higher-income groups seeing income experience little to no effect on decline. For example, within the lowest annual household income category (less than USD 20,000 a year), PFQoL was 3.10 (SE = 0.74, *p* = 0.003) and 5.22 (SE = 0.97, *p* < 0.001) units higher among women residing in stable and upgrading neighborhoods than those in declining neighborhoods, respectively. No significant differences between stable, upgrading, or declining neighborhoods were observed among participants from higher-income categories. Interaction terms between the neighborhood change and race, ethnicity, neighborhood change, and baseline neighborhood economic status were not statistically significant.

## 4. Discussion

### 4.1. Summary of Key Findings

This study describes the various types of neighborhood changes that have occurred over a critical period in the United States and their association with different HRQoL outcomes in a diverse sample of older women. Compared to neighborhoods classified as declining and stable in our study, upgrading neighborhoods had a significant and positive association with overall PFQoL among our sample, and this association varied significantly between different baseline levels of participants’ household income. For SRQoL, we found no significant associations with neighborhood change in fully-adjusted models.

In interpreting these findings, it is worth noting how the different quality of life measures are constructed: the PFQoL measure used nine items to assess how one’s health limits one’s engagement in specific physical activities, whereas the SRQoL used a single item to assess overall quality of life. Neighborhood changes may present more or fewer attractive opportunities (e.g., increased access to public neighborhood nature, decreased safety) to maintain recommended levels of physical activity that could mitigate declines in physical functioning commonly observed among aging populations [52]. Moreover, research among older adult populations demonstrates how associations between the built environment and physical activity can be moderated by individuals’ perceptions of their neighborhoods (e.g., personal and pedestrian safety, quality, and attractiveness of public space). For example, older adults in one study who perceived their neighborhoods to be less safe were also more likely to misjudge the walking distance to certain neighborhood amenities, like supermarkets [53]. Thus, declining neighborhoods may introduce both real and perceived barriers for physical activity, which pose a particular threat to mobility and healthy aging for older adults.

The relatively small changes observed in our study are not as large as effect estimates of major clinical and psychological conditions on PFQoL [54,55,56]. While coefficients of association for these relationships are relatively small, they were robust even after adjustment for social determinants of health, such as household income, marital status, and employment, which were significantly related to QoL outcomes in all models. For example, disability had the largest association with both PFQoL (−32.79 (−34.15, −31.36)), followed by household income (the highest income level of USD 75,000+ was above the lowest level of <USD 20,000 by 8.70 (7.99, 9.43)) and age (the oldest age group was 8.35 (−8.95, −7.67) below the youngest). Other studies have demonstrated the mean differences in populations with and without chronic medical conditions (e.g., found that dialysis patients reported PFQoL roughly 40 points lower than the general U.S. population) [55]; still, others have documented social PFQoL gradients for women by income and class (e.g., lower income/class associated with worse outcomes) and that these patterns are consistent among women with and without disease [57].

The relatively small effect sizes we observed might be partially explained by the relatively diffuse mechanisms of neighborhood change that might impact upon PFQoL versus bodily conditions that are known to affect mental and physical health. Another possible explanation may be the psychological resources necessary for successful aging, such as resilience, defined as one’s self-reported ability to bounce back from stress [58,59]. Previous studies demonstrate that WHI women report high levels of psychological resilience, which is positively associated with HRQoL-related factors, including engagement in protective health behaviors such as physical activity, and negatively associated with perceived stress [60,61]. Other research suggests that psychological resilience is a significant predictor of HRQoL in older adults, particularly in the psychological domain [62,63]. High levels of psychological resilience in our sample may have contributed to the attenuation of the association between neighborhood changes (potential stress) and SRQoL (the psychological aspect of HRQoL) in our sample; further research is needed.

### 4.2. Previous Evidence on Neighborhood Change and HRQoL 

Our findings are consistent with previous literature suggesting neighborhood changes can have both negative effects (in the case of declining neighborhoods) and positive effects (in the case of upgrading neighborhoods) on residents. Although the specific ways in which upgrading and declining may impact HRQoL are understudied, we used previous research to synthesize and discuss potential mechanisms. In our study, neighborhood change (as measured by household median income) may have affected HRQoL (particularly physical functioning) by altering the built environment and perceived access to health-promoting resources as well as by affecting the community social environment [64].

Landis additionally described a neighborhood change classification of “gentrifying”, defined as upgrading tracts in the lower two deciles at baseline. Given our interest in modeling the effect of neighborhood changes, we excluded participants in deciles that could not register both upward or downward change owing to ceiling/floor effects (e.g., bottom two decile tracts could not be classified as “declining”). Still, the question of gentrification remains critical to many public policy debates and is worth exploring further in future research. For instance, upgrading can positively affect the placement and upkeep of community resources vital to health, such as neighborhood parks and open spaces that encourage physical activity or supermarkets that supply high-quality foods. Yet, the pace and magnitude of upgrading could also matter economically (e.g., tenants might be pushed out with increasing rents, higher-end retailers might replace those that cater to lower-income populations) and socially (e.g., displacement of lower socioeconomic status [SES] individuals and replacement with higher SES individuals). Landis’ study of neighborhood socioeconomic changes from 2000–2010 suggested that neighborhood upgrading or decline contribute to displacement although the effects are “far from systematic”, with variables such as median age and household composition also explaining much of the observed residential turnover [65]. Gentrification can also negatively affect community social cohesion when long-time residents, integral members of social networks of trust and support, may be less accessible and place seniors at risk of social isolation [66].

### 4.3. Key Race and Income Findings 

Previous studies have suggested that neighborhood change can either improve or harm communities and levels of HRQoL among residents based (at least partially) on race, ethnicity, and income [67]. While interaction models did not reveal significant differences by race or ethnicity, we did find a significant income interaction effect, whereby the negative relationship between neighborhood decline and PFQoL was strongest among lower-income groups. Conversely, PFQoL had no relationship to neighborhood change among the highest income groups in our study. While we did not find any significant interaction effects with race, descriptive statistics indicate that a disproportionately high number of Black/African American women lived in declining neighborhoods relative to White and Asian participants.

Living in a lower socioeconomic status neighborhood has been associated with greater perceived neighborhood strain (stress), which, in turn, has been associated with poorer physical functioning [64,68]. One study of breast cancer survivors found negative associations between neighborhood poverty rates and physical functioning to be fully explained by physical activity and body mass index [69]. As with the case of gentrification, the mechanisms underlying this relationship deserve further attention; previous research has suggested that even if health-promoting resources are introduced to a previously low-income community, low-income residents may be less likely to use the resources due to the simultaneous changes in their social and psychological conditions (including less social integration, perceived control, and greater financial strain) compared to high-income residents [64,70].

Neighborhood social environment factors, such as the percentage of people with incomes below the poverty level in a census tract or block group, have been linked to all-cause mortality, cause-specific mortality, coronary heart disease, perceived poor health, and cardiovascular risk behaviors and poorer HRQoL, including physical functioning [16]. Furthermore, a high level of perceived neighborhood problems (e.g., racial discrimination), which may arise from the destabilizing effects of neighborhood change and may be associated with poorer HRQoL, poorer physical functioning, and increased depressive symptoms among vulnerable populations [16]. In all of these examples, the question of accumulated exposure to disadvantage remains key to more nuanced understandings of how individual elements of neighborhood change may result in adverse health outcomes.

### 4.4. Strengths and Limitations 

#### 4.4.1. Limitations

Several limitations should be noted. Census data were used to examine the neighborhood changes using an adapted “double decile difference” method, which was initially proposed for its ability to conservatively identify significant shifts in neighborhood median income relative to a particular metro area. Owing to the structure of our data, we used a larger geographic scale (U.S. census regions) to rank tracts and furthermore only ranked those tracts where participants resided, which may limit the transferability of the method. However, the large number of WHI participants, with representation across a variety of socioeconomic and neighborhood factors, and the conservative nature of the classification system (only decile changes > 1 are counted) mitigate some of these concerns. 

To account for possible floor/ceiling effects, we also excluded participants in the lowest/highest deciles of neighborhood median household income at baseline. While this ensures that all included participants have three possible neighborhood change trajectories (i.e., declining, stable, and upgrading), it likely understates the important effects of persistently disinvested or privileged areas although these are at least partially accounted for by the included lower- and higher-decile neighborhoods classified as stable.

Additionally, the areas where our participants resided may have been somewhat localized to the 40 nationwide WHI recruiting sites. Therefore, urban/rural divides (i.e., WHI had more urban recruiting sites than rural ones) and potential recruitment barriers (i.e., access to study sites, compensation, parking, public transit) may have indirectly precluded the most vulnerable groups of women from participating in WHI.

As in many neighborhood studies, the role of self-selection bias may also be present. Participants may have intentionally self-selected into a certain type of neighborhood (i.e., upgrading). Further, we do not know how long individuals in the non-mover sample have lived in their neighborhood or whether that neighborhood was already undergoing change prior to the 2000 census. Relatedly, historical and contemporary injustice, such as structural racism, limit the residential opportunities available to women of color in the U.S., particularly within low-income populations. This unjust limitation operates through widespread policies and practices at national and local levels, such as redlining or racially restrictive covenants [71]. The degree to which racism intersects with other forms of oppression (e.g., ageism, ableism) and how these factors underlie the neighborhood conditions experienced by the participants in our study is unknown although other studies have shown the far-reaching effects of discriminatory policies on present-day wealth and well-being, particularly within the African American community [72,73,74,75]. In sum, we recognize that our participants have not been “allocated” to neighborhoods at baseline; instead, many have likely ended up in particular places as a result of past and present inequities and racism.

Additionally, a large proportion (41%) of WHI participants appear to have moved during the period we assessed and were excluded from these analyses. While it is difficult to say whether this rate of moving is representative of older adult women more generally, the Panel Study of Income Dynamics estimated that 38% of adults aged 50 and above moved between 2001 and 2011 and that while the likelihood of moving generally decreases over the life course, it tends to rise in older age, as individuals may relocate to assisted living facilities [49,76,77]. Future research could separately unpack the influence of moving from one neighborhood context to another, as the possible mechanisms and confounders implied in these kinds of analyses could be quite distinct from those in this study. Given these considerations, our findings—while novel and important for public health and aging in place among older women—they may not be generalizable to other populations (i.e., younger, transient, men).

In future studies, it will be important to further explore the impact of moving from one neighborhood to another among women who moved during the course of the study, the potential confounding effects (non-psychological (e.g., marital status) and psychological resilience resources), and whether relationships between neighborhood change and quality of life are mediated by lifestyle and psychosocial variables. Furthermore, studies that are better powered to examine subgroup differences by race and income are needed to validate the interaction analyses we performed. Future explorations into ethnicity may also be warranted, as recent research by Garcia and colleagues illustrated somewhat counter-intuitive positive associations between neighborhood socioeconomic position and adverse health outcomes (in their studies, progression of type 2 diabetes) [78,79].

#### 4.4.2. Strengths

Our study fills a gap in the literature that has individual, community, and policy implications. To the best of our knowledge, this is among the first longitudinal studies to examine neighborhood change and quality of life outcomes in older women nationwide with a large and diverse sample. Given the political and scientific context of WHI—a massive longitudinal study funded in part to counteract prevailing male-dominated health research paradigms—and the known housing market disadvantages for women, this study also centers women’s experiences and needs [38,80]. Additionally, our use of a self-reported measure of perception further grounds the study in the individual experiences of women.

Furthermore, the study’s time period at least partially includes major upheavals to many communities across the United States, owing to the housing market crisis of 2008. Importantly, WHI’s 76 clinical centers, remote sites, and satellite clinics span the rural-suburban–rural continuum in the U.S. and recruited study participants representing a diversity of influential social determinants of health. Thus, this study provides a unique and high-level perspective on neighborhood change and quality of life among an aging population. Taken together, our findings call attention to the complexity of neighborhood change and the need for more research that examines how the intersection of neighborhood change and individual-level HRQoL is experienced differently by race and income as well as the effects of neighborhood changes on older women who are aging in place. 

## 5. Conclusions

It is likely that a substantial portion of WHI participants have witnessed major changes in their neighborhoods over the past two decades. Gaining a better understanding of how neighborhood changes may have affected study participants can provide important environmental context for their current health status and inform future behavioral interventions within and beyond the WHI. Here, we find that in addition to well-documented social determinants of health and static neighborhood SES, neighborhood changes that individuals experience are also significantly related to facets of their quality of life. These findings provide further evidence on the deleterious effects of neighborhood decline and how lower-income individuals experience these effects even more strongly than those with higher incomes. Healthy aging stakeholders and advocates (psychologists, nonprofit and community organizations, and educators) should note these additional dimensions of vulnerability as they consider ways to support the physical and mental health needs of women aging in changing neighborhoods.

## Figures and Tables

**Table 1:** Characteristics of study population by neighborhood change classification.

	Stable $-1\geq \Delta_{decile}\;\leq 1$	Declining $\Delta_{decile}\;\leq 2$	Upgrading $\Delta_{decile}\;\geq 2$	*p*-Value *
Count (*n*, %)	37,879 (76.9)	6022 (12.2)	5353 (10.9)	<0.001
Census tract median household income at baseline (mean, SD)	USD 53,304 (11,272)	USD 53,421 (9992)	USD 49,172 (9575)	<0.001
Age group, *n* (%)				0.043
50 to 54 (ref.)	4902 (12.9)	798 (13.3)	741 (13.8)	
55 to 59	8045 (21.2)	1220 (20.3)	1083 (20.2)	
60 to 69	18,193 (48.0)	2984 (49.6)	2552 (47.7)	
70 to 79	6739 (17.8)	1020 (16.9)	977 (18.3)	
Race				<0.001
American Indian/Alaskan Native	77 (0.2)	15 (0.2)	14 (0.3)	
Asian	1362 (3.6)	126 (2.1)	231 (4.3)	
Black/African American	2430 (6.4)	816 (13.6)	408 (7.6)	
Native Hawaiian/Other Pacific Islander	68 (0.2)	2 (0.0)	5 (0.1)	
White	32,882 (86.8)	4889 (81.2)	4551 (85.0)	
More than one race	484 (1.3)	68 (1.1)	64 (1.2)	
Unknown/Not reported	576 (1.5)	106 (1.8)	80 (1.5)	
Ethnicity				0.236
Hispanic	1514 (4.0)	224 (3.7)	221 (4.1)	
Not Hispanic	36,107 (95.3)	5765 (95.7)	5086 (95.0)	
Unknown/Not reported	258 (0.7)	33 (0.5)	46 (0.9)	
Disability status				0.275
No self-reported disability (ref.)	35,206 (92.9)	5593 (92.9)	4984 (93.1)	
Self-reported disability	490 (1.3)	90 (1.5)	55 (1.0)	
Unknown	2183 (5.8)	339 (5.6)	314 (5.9)	
Household income				0.013
Less than USD 20,000 (ref.)	4480 (11.8)	705 (11.7)	693 (12.9)	
USD 20,000 to USD 34,999	8906 (23.5)	1416 (23.5)	1246 (23.3)	
USD 35,000 to USD 49,999	8180 (21.6)	1330 (22.1)	1094 (20.4)	
USD 50,000 to USD 74,999	7911 (20.9)	1275 (21.2)	1078 (20.1)	
USD 75,000 or more	5910 (15.6)	885 (14.7)	910 (17.0)	
Unknown	2492 (6.6)	411 (6.8)	332 (6.2)	
Educational attainment				<0.001
High school diploma, GED, or less	9054 (23.9)	1323 (22.0)	1181 (22.1)	
Vocational or training school	4084 (10.8)	650 (10.8)	546 (10.2)	
Some college or associate degree	10,423 (27.5)	1737 (28.8)	1403 (26.2)	
College graduate or baccalaureate degree	4004 (10.6)	651 (10.8)	598 (11.2)	
Some post-graduate or professional degree	4094 (10.8)	677 (11.2)	637 (11.9)	
Master’s or doctoral degree	5925 (15.6)	943 (15.7)	961 (18.0)	
Unknown	295 (0.8)	41 (0.7)	27 (0.5)	
Employment status				0.902
Employed (ref.)	22,026 (58.1)	3529 (58.6)	3136 (58.6)	
Not employed	13,670 (36.1)	2154 (35.8)	1903 (35.5)	
Unknown	2183 (5.8)	339 (5.6)	314 (5.9)	
Marital status				<0.001
Married or in marriage-like relationship (ref.)	25,723 (67.9)	3885 (64.5)	3469 (64.8)	
Never married	1491 (3.9)	252 (4.2)	282 (5.3)	
Divorced or separated	4855 (12.8)	895 (14.9)	763 (14.3)	
Widowed	5654 (14.9)	953 (15.8)	815 (15.2)	
Unknown	156 (0.4)	37 (0.6)	24 (0.4)	
Census region				<0.001
Northeast	9400 (24.8)	1031 (17.1)	978 (18.3)	
South	9510 (25.1)	1679 (27.9)	1485 (27.7)	
Midwest	7692 (20.3)	1817 (30.2)	1396 (26.1)	
West	11,277 (29.8)	1495 (24.8)	1494 (27.9)	

* *p*-values from chi-square distributions for categorical variables and linear regression model for continuous census tract median household income variable.

**Table 2:** Average quality-of-life outcomes by neighborhood change classification.

Outcome	Stable Mean (SD)	Declining Mean (SD)	Upgrading Mean (SD)	*p*-Value *
Self-rated quality of life (SRQoL)	7.97 (1.60)	7.97 (1.61)	8.00 (1.59)	<0.001
Physical functioning-related quality of life (PFQoL)	73.1 (25.4)	72.9 (25.6)	74.3 (25.0)	<0.001

* Note: *p*-values calculated via ANOVA (type II tests).

**Table 3:** Point Estimates and 95% Confidence Intervals for Quality-of-Life Outcomes and Change Relative to Stable Neighborhoods for Unadjusted and Fully-Adjusted Models.

Outcome	Model	Covariates	Declining ^a^ Estimate (95% CI) SE, *p*-Value	Upgrading ^a^ Estimate (95% CI) SE, *p*-Value	Variance Components ^a^ σ^2^, ICC ^b^ Marginal R^2^/Conditional R^2^
Self-rated quality of life (SRQoL)	0	Time + study design	0.02 (−0.02, 0.05) 0.16, 0.307	0.02 (−0.01, 0.06) 0.17, 0.261	1.01, 0.60 0.034/0.615
	3	Model 0 + neighborhood change + sociodemographic characteristics + tract median income decile at baseline	0.02 (−0.01, 0.05) 0.16, 0.168	0.03 (0.00, 0.06) 0.17, 0.117	1.01, 0.59 0.059/0.615
Physical functioning-related quality of life (PFQoL)	0	Time + study design	−0.37 (−0.74, 0.31) 0.26, 0.146	1.00 (0.50, 1.58) 0.27, <0.001	142.54, 0.76 0.086/0.783
	3	Model 0 + neighborhood change + sociodemographic characteristics + tract median income decile at baseline	−0.46 (−0.79, 0.15) 0.25, 0.059	1.18 (0.68, 1.82) 0.26, <0.001	142.44, 0.74, 0.132/0.777
	4	Model 3 + interaction with participant household income	−3.10 (−4.79, −1.83) ^c^ 0.74, <0.001	2.12 (0.43, 3.28) ^c^ 0.74, 0.004	142.44, 0.74 0.133/0.777

^a^ SE, standard error; CI, confidence interval. ^b^ σ^2^, residual variance; ICC, intraclass correlation. ^c^ Main effect of neighborhood change. Significant interaction term between neighborhood change variable and participant’s household income at baseline reveals that these differences were mainly driven by lower-income participants; differences between neighborhood change categories among higher-income participants were not significant.

## Data Availability

For further information about WHI Data Files, please visit: https://www.whi.org/page/working-with-whi-data (accessed on 13 April 2022).
